# *iMeta*: Boosting academic sharing and collaboration via social media

**DOI:** 10.1002/imt2.70085

**Published:** 2025-10-09

**Authors:** Xiaofang Yao, Jiqiu Wu, Tengfei Ma, Chun‐Lin Shi, Canhui Lan, Danyi Li, Jingyuan Fu, Ziang Shen, Tong Chen, Yong‐Xin Liu

**Affiliations:** ^1^ Genome Analysis Laboratory of the Ministry of Agriculture and Rural Affairs, Agricultural Genomics Institute at Shenzhen Chinese Academy of Agricultural Sciences Shenzhen China; ^2^ APC Microbiome Institute University College Cork Cork Ireland; ^3^ Department of Genetics, University Medical Center Groningen, University of Groningen Groningen The Netherlands; ^4^ State Key Laboratory of Herbage Improvement and Grassland Agro‐ecosystems, Centre for Grassland Microbiome, College of Pastoral Agriculture Science and Technology, Lanzhou University Lanzhou China; ^5^ ANGENOVO Oslo Norway; ^6^ Ningbo R‐Institute of Science Communication Ningbo China; ^7^ School of Life Science and Technology Wuhan Polytechnic University Wuhan China; ^8^ Department of Pediatrics, University Medical Center Groningen, University of Groningen Groningen The Netherlands; ^9^ School of Biomedical Sciences and Engineering South China University of Technology Guangzhou China; ^10^ State Key Laboratory for Quality Ensurance and Sustainable Use of Dao‐di Herbs, National Resource Center for Chinese Materia Medica China Academy of Chinese Medical Sciences Beijing China

## Abstract

Social media platforms have revolutionized scientific communication by bridging gaps between researchers, academic journals, and global audiences. This article showcases *iMeta*, an open‐access journal that leverages a diversified social media framework to enhance bilingual dissemination, boost full‐text downloads, and amplify international influence. Since its editorial board founded, *iMeta* has achieved a series of milestones: integrating platforms like WeChat, Bilibili, X (formerly Twitter), YouTube, and BlueSky; launching *iMeta*‐branded journals *iMetaOmics* and *iMetaMed*; and being indexed in prominent databases including PubMed, SCIE, and ESI. As of August 2025, the journal has recorded 1,334,761 full‐text downloads and 10,560 total citations, with a 2024 impact factor of 33.2. A significant positive correlation between downloads and citations highlights how strategic social media integration and *iMeta*'s growth drive visibility and influence, positioning it as a leading journal in its field.

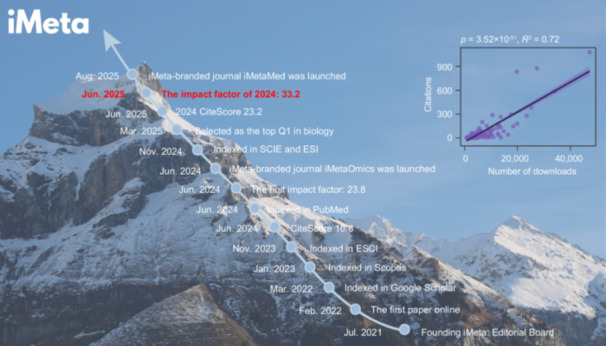

Academic journals have long served as essential platforms for disseminating scientific knowledge and presenting research findings. However, promoting journals' high‐quality visibility and global influence is challenging due to singular dissemination channels and regional language barriers. The obstacles hinder our capacity to obtain journal content and frontier scientific insights, in turn restricting the spread of journals' academic influence. The rapid rise of social media has reshaped this landscape: it has transformed how people communicate, access information [1], and engage with scientific content [2]. Social media platforms such as WeChat, Bilibili, X (formerly Twitter), and YouTube have approximately 1 billion, 341 million, 145 million, and 122 million daily active users, respectively [3]. Many individuals share their personal experience and research activities on these platforms [4]. Diversified social media platforms have become essential for researchers to enhance collaborations, stay updated on scientific advancements, and access research opportunities [1], making high‐quality development of journals closely tied to effective use of these tools. To address the journal dissemination challenges, *iMeta* journal employs an innovative strategy featuring video abstracts, bilingual publications, and multimedia social media outreach.

## *iMeta*: An innovative strategy for enhancing research journal visibility

**Figure 1:**
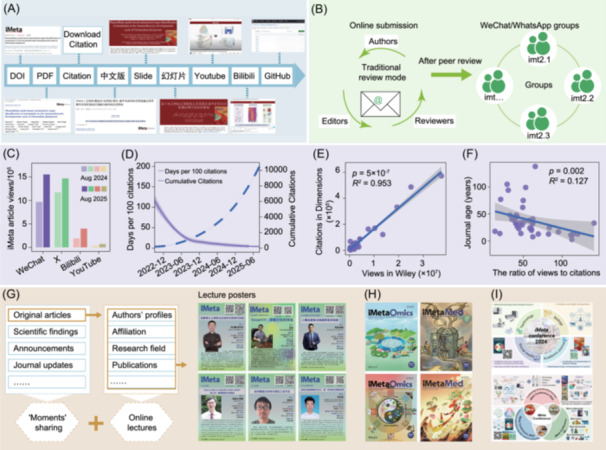
Social media platforms play essential roles in the development of *iMeta*. (A) Promoting research dissemination via diverse strategies, including disseminating Chinese‐English bilingual articles and related multimedia (slides, videos) across platforms like WeChat, Bilibili, YouTube, and so forth. (B) Accelerating article revision and polishing through WeChat/WhatsApp groups. After preliminary accepted, the average period for revising and polishing articles is reduced from 2 months to less than 1 month. (C) *iMeta* article views on WeChat, X (formerly Twitter), Bilibili, and YouTube in 2024 and 2025. (D) Days per 100 citations and cumulative citations over time. (E) Correlation between citations in Dimensions and views in Wiley Online Library. (F) Correlation between journal age and the ratio of views to citations. (G) WeChat “Moments” sharing and online lectures, featuring content like original articles, scientific findings, and author profiles. (H) *iMeta*‐branded journals *iMetaOmics* and *iMetaMed*. (I) *iMeta* conferences held in 2024 and 2025.

*iMeta* is a comprehensive open‐access journal, launched in 2022 in partnership with Wiley by the microbiology and bioinformatics research community. It aims to publish high‐quality papers for a broad and diverse audience, with a focus on disciplines such as metagenomics, biotechnology, and bioinformatics [5]. *iMeta* has emerged as a model for how emerging journals can rapidly build influence by embracing innovative communication strategies. Central to its approach is leveraging diversified social media platforms, such as WeChat (ID: iMetaScience), Bilibili (space.bilibili.com/505919932), X (x.com/iMetaScience), YouTube (youtube.com/@imetascience9128), and BlueSky (bsky.app/profile/imetascience.bsky.social). In addition, *iMeta* also employs a variety of content formats, including bilingual contents, author explanation videos, and AI‐generated summary videos, and other formats to introduce articles in an accessible and engaging manner (Figure 1A). This multifaceted approach has significantly accelerated the dissemination of its publications and broadened its audience base. As of August 2025, *iMeta* has been downloaded 1,334,761 times and obtained 10,560 citations.

## Leveraging X and WeChat to amplify journal visibility and impact

In the current digital era, social media has become an indispensable tool for academic journals aiming to expand their visibility and global reach. An extensive survey of 13,826 journals indexed by the Web of Science revealed that journal presence on social media platforms such as X and Facebook varies between 7.1% and 14.2% across disciplines [6]. Leading international journals, such as *Nature, Science*, and *Cell*, have actively embraced social media, having posted approximately 83,000, 44,600, and 12,600 tweets on X, respectively. Their impact is further evidenced by massive followers, with *Nature* and *Science* amassing over 2.6 million and 4.4 million followers, respectively (Aug 2025).

Importantly, these journals are also investing in platform‐specific strategies for broader engagement in non‐English regions. WeChat has emerged as a popular tool for science communication in China and among global Chinese researchers. In a 2019 Springer Nature survey, 94% of the 528 researchers in China had used WeChat in a professional context [3]. Recognizing the influence of WeChat in the Chinese scientific community, *Science*, *Cell* and *Nature* launched official WeChat accounts in 2017, 2018, and 2021, respectively. To ensure localized engagement, they even established offices in China to manage and operate these accounts. Additionally, a nationwide analysis of China journals revealed that 65.3% of journals indexed in the Chinese Social Sciences Citation Index had established WeChat official accounts, which collectively published over 193,000 original posts in 2022 [7]. These official WeChat accounts serve as critical nodes for knowledge dissemination and academic branding.

Taking *iMeta* as an example, since its official launch, its WeChat official account persistently publishes daily updates of newly released articles. *iMeta* now has 59,348 subscribers and has posted 991 contents, which have obtained 5.45 million views. Notably, these contents include 304 original scientific articles, with a combined total of 927,589 views, averaging over 3000 views per article. Beyond its main official account, its contents were also posted through other partners WeChat accounts such as meta‐genome, RXCYJY, and Bio_data, resulting in a combined total of 628,593 views. The first article in *iMeta* titled *ImageGP: An easy‐to‐use data visualization web server for scientific researchers* [8] received an impressive 32,868 views and 378 citations. These data underscore how strategic use of WeChat can elevate a journal's academic influence and visibility.

## Accelerating the revision and polishing after paper acceptance through instant communication

Conventionally, manuscript tracking relies heavily on the static journal portal updates and delayed email notifications, leading to information gaps that persist for days or even months. This system delay forces authors into passive waiting when needing reviewers' comments. Additionally, clarifying ambiguous comments, supplementing data, or adjusting visuals requires time‐consuming email exchanges, significantly delaying manuscript processing. To overcome these inefficiencies, *iMeta* has introduced a more dynamic, interactive efficient collaboration model by integrating WeChat/WhatsApp groups into postacceptance editorial workflow (Figure 1B). Upon article acceptance, an exclusive WeChat group is established for each article. The group not only includes core members of the author team and the editors but also incorporates a professional media operation team. The trinity structure has completely broken the barriers of traditional communication.

Operationally, this system delivers multifaceted advantages. Editors instantly disseminate editor comments within the group. If authors have doubts about a certain comment, they can chat instantly with editors, avoiding information attenuation inherent in email chains. For visual refinements such as chart clarity or legend standardization, the media team may share targeted formatting guidance with examples in the group. After authors revise and upload preview images, all parties can confirm them in the group together, avoiding ineffective repeated revisions that fail to meet requirements. This instant and transparent collaboration builds a partnership, turning academic publishing from an impersonal digital transaction to human‐centered dialogue and adding real‐time warmth to revisions.

## Enhancing global engagement with scientific content through diversified social media platforms

*iMeta* strategically leverages mainstream social media platforms to broaden its international visibility and promote global academic exchange. Platforms such as WeChat, X, Bilibili, and YouTube have played key roles in the journal's outreach efforts, garnering 969,401, 1,174,101, 191,469, and 30,850 views in August 2024, and 1,556,182, 1,472,073, 419,221, and 72,182 views in August 2025, respectively (Figure 1C). Notably, the article views on WeChat official accounts surpassed that on X. However, X remains a widely used platform in the scientific community [3]. *iMeta* official X account (@iMetaScience) has posted 1621 tweets and has more than 2833 followers. Its most popular tweet highlighted EasyAmplicon, an easy‐to‐use, open‐source, reproducible microbiome amplicon pipeline [9], with over 42,500 views. Bilibili and YouTube serve as prominent video sharing platforms within Chinese‐ and English‐speaking communities, respectively [10]. The *iMeta* team, in collaboration with the article authors, created a concise 5‐min video summaries, available in both Chinese for Bilibili and English for YouTube. The average number of views per article on the two platforms is 1541 and 258, respectively. Notably, the highest viewed video on these two platforms were 18,000 and 5052, respectively. The multimedia approaches made research findings more visible, accessible, and easier to understand for relevant scientists. For those seeking a deeper understanding, direct links to the full‐text articles are provided for comprehensive reading.

Critically, enhanced visibility is translating into academic impact. Prior research have showed that journal activity on WeChat is positively correlated with their citations [7]. As of August 2, 2025, articles published in *iMeta* had received a total of 10,500 citations, with an average of 32.95 citations per article. The time interval to achieve each additional 100 citations has ranged from as long as 193 days to as short as 2 days (Figure 1D). The journal's growth of citations demonstrates how integrating publishing with social media accelerates scientific progress. One outstanding article, *Complex Heatmap Visualization* [11], has been cited 1079 times and leads with the highest number of downloads at 45,241. Meanwhile, the other eight most‐cited papers each have over 200 citations and more than 10,000 downloads, linking high‐quality work to broad impact and engagement. Since citation counts remain a key metric of journal impact, these data underscore the growing academic influence of *iMeta*. Furthermore, by fostering public engagement, these platforms contribute to extend research's reach and societal relevance [12].

We further retrieved data on the 2024 full text views, citations for 40 Wiley cooperative journals via Wiley Online Library and Dimensions. The analyses showed a positive correlation between views and citations, and a negative correlation one between the journal age and views to citation ratio (Figure 1E,F). The average ratio of views to citations across these 40 journals was approximately 58, with that of *iMeta* reaching 113. These findings indicate that higher views can increase article citations, and this trend is more pronounced for journals established earlier (Figure 1F).

## Strengthening academic discussion and collaboration

*iMeta* builds interactive communities to foster academic collaboration through timely discussions and insight sharing. These communities serve as dynamic platforms for sharing published original articles, significant scientific findings, conference announcements, and journal updates. Each article is accompanied by detailed profiles of authors, covering their affiliation, title, research field and contribution, publications, received grants, and so forth. *iMeta* also broadens its readership through WeChat “Moments” sharing, a feature similar to Facebook, where users share updates with their network (Figure 1G). This personalized presentation enhances author visibility and helps promote academic networking and potential collaboration. In addition, the *iMeta* team regularly organizes themed online lectures aiming to address trending topics of interest to the research community (Figure 1G). The lectures are steamed live and later made available on multiple social media platforms. Moreover, *iMeta* launched the branded journals *iMetaOmics* [13] and *iMetaMed* [14] (Figure 1H), and held conferences in 2024 [15] and 2025 respectively (Figure 1I). These initiatives have attracted the attention of numerous scientists and significantly increased *iMeta*'s exposure. *iMeta* enhances scientific communication and fosters cross‐disciplinary collaboration through community engagement, author profiles, and academic events, significantly expanding its influence.

## Challenges and perspectives

Several key challenges currently hinder the realization of truly global and inclusive scientific communication. The fragmentation of social media ecosystems across different regions impedes consistent and effective outreach, while linguistic and cultural barriers often limit meaningful engagement with international audiences. Furthermore, maintaining scientific accuracy while adapting content for social media requires a careful balance. Significant differences in user demographics, content preferences, and algorithmic visibility across platforms also necessitate tailored strategies rather than a uniform approach. Finally, assessing the tangible impact of dissemination efforts on academic influence and real‐world collaboration remains methodologically complex. Overcoming these challenges is crucial to fully unlocking the potential of digital science communication.

Looking forward, the rise of diverse social media platforms has opened new frontiers for journal dissemination, promoting real‐time interaction and greater visibility. To leverage these opportunities, *iMeta* will implement a multi‐channel communication strategy involving global platforms—such as WeChat, Bilibili, X, YouTube, and BlueSky—as well as regional Chinese platforms including Douyin, Xiaohongshu, and Zhihu. These initiatives are designed to share high‐quality research and scientific breakthroughs with a broad and diverse global readership. In the future, *iMeta* also plans to expand its reach by launching specialized journals such as *iMetaOmics* and *iMetaMed*, hosting conferences in life sciences and medicine, and organizing English‐language lectures to strengthen global research connections. By integrating localized innovation with a worldwide vision, *iMeta* is poised to become a truly international journal while upholding academic integrity and rigor. We hope that our multi‐platform, bilingual communication model will offer a valuable example for journals aiming to enhance their global influence.

## AUTHOR CONTRIBUTIONS

**Xiaofang Yao**: Conceptualization; investigation; writing—original draft; writing—review and editing; visualization; methodology; formal analysis; data curation. **Jiqiu Wu**: Investigation; writing—review and editing. **Tengfei Ma**: Conceptualization; investigation; writing—review and editing. **Chun‐Lin Shi**: Investigation; review and editing; validation. **Canhui Lan**: Methodology; validation; review and editing. **Danyi Li**: Investigation; review and editing; visualization; validation. **Jingyuan Fu**: Review and editing; validation. **Ziang Shen**: Validation; investigation; data curation. **Tong Chen**: Investigation; validation. **Yong‐Xin Liu**: Conceptualization; funding acquisition; writing—review and editing; data curation; validation; methodology; project administration; supervision; resources. All authors have read the final manuscript and approved it for publication.

## CONFLICT OF INTEREST STATEMENT

Jingyuan Fu is the Editor‐in‐Chief of iMeta; Canhui Lan, Danyi Li, Tong Chen, and Yong‐Xin Liu are Executive Editors of iMeta. The other authors have declared no competing interests.

## ETHICS STATEMENT

No animals or humans were involved in this study.

## Data Availability

The data and scripts used are saved in GitHub https://github.com/yaoxiaofang1/iMetaeditorial/tree/master. Supplementary materials (graphical abstract, slides, videos, Chinese translated version and update materials) may be found in the online DOI or iMeta Science http://www.imeta.science/.
